# Effects of dietary nanoliposome‐coated astaxanthin on haematological parameters, immune responses and the antioxidant status of rainbow trout (*Oncorhynchus mykiss*)

**DOI:** 10.1002/vms3.1461

**Published:** 2024-04-22

**Authors:** Mojdeh Besharat, Houman Rajabi Islami, Mehdi Soltani, Seyed Abdolmajid Mousavi

**Affiliations:** ^1^ Department of Fisheries, Science and Research Branch Islamic Azad University Tehran Iran; ^2^ Department of Aquatic Animal Health, Faculty of Veterinary Medicine University of Tehran Tehran Iran; ^3^ Centre for Sustainable Aquatic Ecosystems, Harry Butler Institute, School of Veterinary and Life Science Murdoch University Murdoch Australia; ^4^ Department of Animal Science, Varamin‐Pishva Branch Islamic Azad University Varamin Iran

**Keywords:** antioxidant, astaxanthin, immunity, nanoliposome, rainbow trout

## Abstract

**Background:**

Astaxanthin is the most prevalent carotenoid in the marine environment and is widely used as an additive in formulated aquafeeds.

**Objectives:**

A 60‐day feeding trial was conducted to consider the effect of dietary nanoliposome‐coated astaxanthin (NA) on haematological parameters, serum antioxidant activities and immune responses of rainbow trout, *Oncorhynchus mykiss*.

**Methods:**

A total of 450 healthy fish weighing 31.00 ± 2.09 g were randomly assigned in triplicate (30 fish per replicate) to 5 dietary treatments: 0 (control), 25.00, 50.00, 75.00, and 100.00 mg kg^−1^ NA.

**Results:**

Fish fed the diet supplemented with 50.00 mg kg^−1^ NA exhibited the highest values of red blood cells, white blood cells, haemoglobin and haematocrit of 1.64 ± 0.01 × 10^6^ mm^−3^, 5.54 ± 0.21 × 10^3^ mm^−3^, 8.73 ± 0.24 g dL^−1^ and 46.67% ± 0.88%, respectively, which were significantly higher than those fed the basal diet (*p* < 0.05). The lowest and highest percentages of lymphocytes (67.67% ± 0.33%) and neutrophils (27.33% ± 1.20%) were also obtained in fish fed 50.00 mg kg^−1^ NA compared to those fed the basal diet (*p *< 0.05). Fish receiving diet supplemented with 50.00 mg kg^−1^ NA revealed the highest serum activity in superoxide dismutase, catalase, glutathione peroxidase, lysozyme and alternative complement and the lowest level of total cholesterol, cortisol, aspartate aminotransferase and alanine aminotransferase than fish receiving the basal diet (*p *< 0.05). Serum immunoglobulin (Ig) and ACH50 contents significantly increased with increasing dietary NA supplementation to the highest values of 43.17 ± 1.46 and 293.33 ± 2.03 U mL^−1^, respectively, in fish fed diet supplemented with 50 mg kg^−1^ NA (*p *< 0.05).

**Conclusions:**

Supplementation of NA in rainbow trout diet at 50 mg kg^−1^ exhibited a positive effect on haematological parameters, antioxidant capacity and immune responses. Administration of such dosage can enhance rainbow trout immune responses against unfavourable or stressful conditions, for example disease outbreaks, hypoxic condition, thermal stress and sudden osmotic fluctuations, which usually happen in an intensive culture system.

## Graphical abstract

This study investigated the effects of dietary nanoliposome coated astaxanthin (NA) on the haematological parameters, immune responses and antioxidant status of rainbow trout. Results showed that increasing levels of NA in the fish diet led to significant improvements in red blood cell count, haemoglobin concentration and white blood cell count, indicating enhanced haematological parameters. Additionally, the study demonstrated that NA positively influenced the antioxidant capacity and immune responses of the rainbow trout.

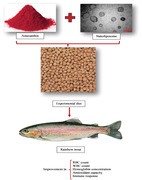

## 1 INTRODUCTION

Astaxanthin is the most prevalent carotenoid in the marine environment and is widely used as an additive in formulated aquafeeds to improve muscle coloration and collagen synthesis, especially in salmon and shrimp (Lim et al., 2018; Lu et al., 2021). Astaxanthin has also been considered a powerful antioxidant that protects the cells from oxidative stress by donating electrons and neutralizing free radicals to form a non‐reactive product (Dose et al., 2016; Muthuraman et al., 2021). Furthermore, dietary supplementation with astaxanthin was found to enhance antibody production, reduce humoral immune responses and modulate the metabolism of diagnostic biomarkers of chronic stress like glucose (Kalinowski et al., 2019; Lim et al., 2019; Lin et al., 2015). The positive effects of astaxanthin on fish growth have also been documented (Liu et al., 2016). The safety of astaxanthin as a dietary supplement in aquafeed was approved by the United States Food and Drug Administration in 1999 (Guerin et al., 2003).

Aquatic animals have principally low ability to biochemically synthesize astaxanthin de novo and thus require it as a part of dietary supplementation for immune‐physiological functions (Basiony et al., 2022; Fang et al., 2019). Although freshwater green microalga *Haematococcus pluvialis* and pink yeast *Phaffia rhodozyma* are the main sources of natural astaxanthin (Niizawa et al., 2021; Zhuang & Zhu, 2021), over 95% of available astaxanthin in the market is produced synthetically (Hu, 2019). It has been reported that the antioxidant activity of natural astaxanthin is 50 times higher than the synthetic forms (Capelli et al., 2013) and at least 10 times higher than that of other carotenoids (Dufossé, 2008). Accordingly, the global demand for natural and more effective astaxanthin products is growing, although high production costs and several technological problems have limited its mass production from natural sources (Villaró et al., 2021).

The rapid growth of the aquaculture industry has increased the need to use astaxanthin as a feed additive (Verlhac Trichet & Amaya, 2022). Historically, astaxanthin is a high‐price feed additive, possessing the most expensive part of feed cost based on the negligible mass fraction in the salmonid feed recipes (Dethlefsen et al., 2016). On the other hand, due to its highly unsaturated molecular structure, this lipophilic pigment has a high sensitivity to various environmental conditions such as heat, light, oxygen and acidity (Martínez‐Delgado et al., 2017; Zhao et al., 2022), which can reduce its biological activities and nutritional availability during aquafeed processing and storage. Therefore, it is extremely necessary to develop new strategies to broaden the storage stability of astaxanthin for application in different industries.

Over recent years, numerous studies have been dealt to improve the stability, solubility and bioavailability of astaxanthin by its embedding encapsulating into natural polymers like chitosan (Liu et al., 2019), cyclic oligosaccharides like hydroxypropyl‐β‐cyclodextrin (Su et al., 2021) or a mixture of protein and carbohydrate (Bassijeh et al., 2020). Among the delivery systems, liposomes and their nanometric versions are the most potential structures for the efficient transport of bioactive agents into the body by either facilitating direct absorption in the target tissue or preventing breakdown by gastric acid (Chariou et al., 2020; Panahi et al., 2017). These amphiphilic vesicles can be synthesized using safe ingredients derived from natural sources, such as soy, milk or eggs (Panahi et al., 2017), making it easier to be approved for employment in food‐grade products.

Although the application of regular liposomes (1–100 µm dimension) is limited due to their large particle size, nanoliposomes (1–100 nm dimension) offer a larger surface area with a higher ability to increase solubility, improve controlled release and enhance the bioavailability of encapsulated materials compared to liposomes (Khorasani et al., 2018; Pan, Wang, et al., 2018). Accordingly, liposomal nanoparticles have been widely used to enhance the nutritional properties of foods in several food industry processes (Liu et al., 2022; Najafi et al., 2022; Sarabandi et al., 2019; Varma et al., 2021; Vijayakumar et al., 2019). However, few studies have been conducted on the use of nanoliposome‐coated astaxanthin (NA) in aquatic animals. In our previous work, the promising effect of dietary NA on the growth performance of rainbow trout has been demonstrated (Besharat et al., 2021). The present study aimed to investigate the effect of different levels of dietary NA on the haematological, immunological and antioxidant properties of rainbow trout.

## 2 MATERIALS AND METHODS

### 2.1 Nanoparticle preparation and physicochemical characterization

Nanoparticle preparation and physicochemical characterization were carried out according to Besharat et al. (2021). Briefly, 5 mg astaxanthin (98% purity, DSM company) was mixed with 200 mg soy phosphatidylcholine and 50 mg cholesterol dissolved in chloroform to form a thin film. After removing chloroform by a rotary evaporator at 50°C, the hydrated sample in 0.05 M phosphate‐buffered solution was vortexed for 30 min at 50°C before being introduced into ultrasonic probe with 5 s pulse‐on and 5 s pulse‐off to obtain nanoparticles. The generated liposome samples were kept in brown glass vials filled with nitrogen and stored at 4°C.

The encapsulation efficiency (EE) and loading capacity (LC) were assessed by mixing an aliquot (400 µL) of NA with 5 mL petroleum ether by gentle agitation for 5 min at 30°C before the mixture was centrifuged at 3000 rpm for 5 min to collect the supernatant. After evaporation of petroleum ether at 50°C, the residual was dissolved in chloroform, and the concentration of free astaxanthin was determined using a UV–vis spectrophotometer (Cary 100 UV‐Vis, Agilent) at 492 nm by employing chloroform as the blank. Each measurement was performed in triplicates. The EE (%) and LC (%) were quantified using the following equations (Pan, Zhang, et al., 2018):

$$\begin{array}{c} EE\left(\%\right)=100\times \left(total\;astaxanthin-free\;astaxanthin\right)/total\;astaxanthin\\ LC\left(\%\right)=100\times \left(total\;astaxanthin-free\;astaxanthin\right)/sample\;weight \end{array}$$

Zeta potential and size distribution of the NA were evaluated in triplicate by the dynamic laser scattering technique using Malvern Zetasizer Nanoseries Nanos ZS90 (Malvern Instruments), where the detector angle was 90°. The nanoparticle morphology was observed with a field emission scanning electron microscope (TESCAN MIRA3, Tescan).

### 2.2 Diet preparation and storage

The basal diet was purchased from a commercial company with the specifications of 40% protein, 14% fat, 10% ash, 11% moisture and 3.5% fibre. The experimental diets were prepared by adding 0.00, 25.00, 50.00, 75.00 and 100.00 mg NA to each kg of the basal diet. Cellulose was replaced with a reduced amount of NA in all experimental diets. The ingredients of each diet were mixed well to create a uniform powder. The powder was then mixed well with an appropriate level of binder (agar) and water to produce a wet dough. The minced dough was made again into pellets by compressing through a perforated disc (2 mm diameter round hole) in a laboratory pelletizing machine without heating. The pellets were dried in an oven at 40°C for 1 h and stored in plastic bags in a refrigerator at 4°C until consumption (Besharat et al., 2021).

### 2.3 Experimental design and sample collection

Healthy rainbow trout (*Oncorhynchus mykiss*, Walbaum 1792) were procured from a commercial fish farm in Mazandaran Province, Iran, and acclimatized to the experimental condition in an indoor recirculating aquaculture system under natural photoperiod. During the acclimation period, fish were manually fed with the basal diet three times a day (9:00, 12:00 and 17:00) at 2%–3% of weight according to the water temperature. Freshwater was supplied in the recirculating system, and aeration was continuously provided via air stones to maintain dissolved oxygen levels near saturation. The water quality conditions were maintained as follows: the water temperature was 11.0 ± 0.2°C, pH was 8–8.2, dissolved oxygen was >11 mg L^−1^ and total hardness was 100–120 mg L^−1^. The NO_2_‐N and NH_4_^+^‐N concentrations were <0.02 and <0.5 mg L^−1^, respectively. The rearing water was renewed daily at the same time with a volume of 30% freshwater.

At the beginning of the experiment, fish with an initial mean weight of 31.00 ± 2.09 g were randomly distributed into 15 cylindrical fibreglass tanks (water volume 1000 L) at 30 fish per tank. Each experimental diet was randomly assigned to a group of three tanks. Experimental fish were fed with the corresponding diets for 60 days using the same procedure as described for the acclimation period in the same recirculating system with similar water quality parameters. The fish weight was recorded every 15 days, and the feeding rate was adjusted based on the fish weight increments. Before biometry, the fish were starved for 24 h and anaesthetized with clove oil extract (150 mg kg^−1^) (Arab & Rajabi Islami, 2015). At the end of the feeding trial, the number and total weight of fish in each tank were counted and weighed for determination of fish growth performance and feed utilization efficiency. Peripheral blood samples of fish were randomly withdrawn with a 1.0 mL syringe from the caudal vein of three fish per tank and immediately divided into two aliquots; one was transferred to the tube containing anticoagulants (40 IU mL^−1^ heparin) for haematological analysis, and the other half was transferred to non‐heparinized tubes for biochemistry, antioxidants and immunological analysis after centrifuging at 3500 × *g* for 10 min at 4°C (Sigma centrifuge 3–30 k, Sigma Laborzentrifugen GmbH) and immediately stored at −80°C until analysis.

### 2.4 Haematological assays

Red blood cells (RBCs, ×10^6^) and white blood cells (WBCs, ×10^3^) were counted using an improved Neubauer haemocytometer after diluting with an appropriate amount of Hayem and Turck solutions, respectively (Farahnak Roudsari et al., 2021). Haematocrit (Hct, %) percentage was analysed in glass capillary tubes using a microhaematocrit centrifuge (MC‐150, Tomy Tech.) within 40 min after blood sampling (Jamalzad Falah et al., 2020). Haemoglobin concentration (Hb, g dL^−1^) was determined based on the cyanomethemoglobin method by measuring a wavelength of 540 nm (Ahmadpanah et al., 2019). The derived blood indices, including mean cell volume (MCV, fL) as the average size of erythrocytes, mean cell haemoglobin (MCH, pg) as the weight of haemoglobin in the average RBC and (MCHC, g dL^−1^) as the relationship between the size of erythrocytes and their haemoglobin contents, were determined using the following formulae (Moazenzadeh et al., 2020):

$$\begin{array}{c} \begin{array}{c} MCV=10\times Hct/RBC\\ MCH=10\times Hb/RBC \end{array}\\ MCHC=100\times Hb/Hct \end{array}$$

Differential leukocyte count was determined by preparing a blood smear from each blood sample, fixing with 96% ethanol for 30 min, and staining with Giemsa for lymphocyte, monocyte, neutrophil and eosinophil count under a compound microscope.

### 2.5 Antioxidant assays

The values of superoxide dismutase (SOD), catalase (CAT), glutathione peroxidase (GPX), aspartate aminotransferase (AST), alanine aminotransferase (ALT) and malondialdehyde (MDA) were tested in sera samples using an automatic biochemical analyser (Prestige 162 24i) by the commercial kits (Pars‐Azmoon). The SOD activity was determined by monitoring its ability to inhibit superoxide anions produced by the xanthine and xanthine oxidase reaction systems at 550 nm (Saheli et al., 2021). One unit of SOD activity was defined as the amount of enzyme yielding a 50% inhibition of the reaction and expressed as IU L^−1^. The CAT activity was determined by measuring the decrease of hydrogen peroxide concentration as the substrate in phosphate buffer at 240 nm. One unit of CAT activity was defined as the amount of enzyme necessary to transform 1 µmol hydrogen peroxide per min at 25°C and expressed as IU L^−1^. The conversion rate of glutathione to oxidized glutathione using the colorimetric method was also used to monitor the activity of GPX at 412 nm after treating with KCN solution (Drabkin's reagent; Sigma Chemical Co.) to prevent the haemoglobin interference (Mostafavi et al., 2022). One unit of GPX was defined as the enzyme level needed to oxidize 1 µmol of NADPH per min and expressed as IU L^−1^. The AST and ALT activities were determined spectrophotometrically by measuring the optical density at 546 nm (Reitman & Frankel, 1957). One unit of AST and ALT was defined as the enzyme level required at 37°C (pH 8.0) to generate 1.0 µmol min^−1^ glutamate and pyruvate, respectively.

Lipid peroxidation level was assessed by measuring the thiobarbituric acid–reactive substance concentrations based on the method described by Ohkawa et al. (1979). Values were reported as the concentration of MDA (µmol L^−1^). The serum cortisol concentration was determined based on the procedure described by Mohammadi and Khara (2015) using a commercial radioimmunoassay (Immunotech Company) and expressed as ng mL^−1^.

### 2.6 Immunological assays

Serum lysozyme activity (Lys) was evaluated according to the method described by Ellis (1990) based on the lysis of the lysozyme‐sensitive Gram‐positive bacterium, *Micrococcus lysodeikticus* (Sigma). Briefly, 50 µL serum was added to a 2‐mL suspension of *M*. *lysodeikticus* (0.2 mg lyophilized cell mL^−1^ sodium phosphate buffer [0.05 M], pH 6.2) and incubated with gentle shaking at 22°C. The change in turbidity was measured at 450 nm after 30 and 180 s with a temperature‐controlled spectrophotometer (Biophotometer D30, Eppendorf). A unit of Lys was defined as the amount of serum causing a decrease in the optical density reading of 0.001 min^−1^.

Serum alternative complementary activities (ACH 50) were evaluated following the procedure described by Yano (1992) using rabbit RBCs (RaRBC) as target cells. Briefly, RaRBCs were washed three times with ethylene glycol tetra‐acetic acid–magnesium–gelatin/veronal buffer (EGTA–MG–GVB), and the number of cells was adjusted to 2 × 10^8^ mL^−1^ using a haemocytometer. Complete lysis (100%) was achieved by resuspending 100 µL RaRBC in 3.4 mL distilled water and measuring the optical density at 414 nm against distilled water as blank to achieve 0.740% lysis. Sera samples were then diluted 100 times with EGTA–MG–GVB, and different volumes were prepared in a sterile test tube, whereas the total volumes reached up to 0.250 µL with the buffer before reacting with 0.1 mL of RaRBC. Finally, the mixture was incubated at 20°C for 90 min with regular shaking and centrifuged at 1600 × *g* for 10 min at 4°C using a Sigma centrifuge (3–30 k, Sigma Laborzentrifugen GmbH) to remove non‐lysed erythrocytes after adding 3.15 mL of the saline solution (0.85% v/v) to each tube. The absorbance of the supernatant was measured at 414 nm using a spectrophotometer (Cary 100 UV–vis, Agilent). The volume of serum inducing 50% haemolysis was used for determining the ACH50 using the following equation:

$$ACH50\;(U\;{mL}^{-1})=1/K\times r\times 0.5$$

where K is the amount of serum inducing 50% lysis, r is the reciprocal of the serum dilution and 0.5 is the correction factor.

Serum immunoglobulin (Ig) content was measured according to Siwicki and Anderson (1993). This assay measures total plasma protein content using a microprotein assay (C‐690; Sigma) before and after precipitation of Ig molecules using a 12% (w/v) solution of polyethylene glycol (Sigma). The difference between the total protein content of the serum sample and the polyethylene glycol–treated sample corresponds to the total Ig content that was expressed as mg dL^−1^.

### 2.7 Statistical analysis

The results were presented as means ± standard error of the mean (SEM) of three replicates. The Shapiro–Wilk test was initially used to check the distribution of data normality. All normal data were subjected to one‐way analysis of variance using SPSS 22.0 software (IBM). Differences among the means were tested by Duncan's multiple range test after checking the homogeneity of variances. The level of significance was chosen at *p* less than 0.05. All statistical analyses were carried out with SPSS cStatistics 22 (IBM). Microsoft Excel 2017 was used to draw graphs.

## 3 RESULTS

### 3.1 Haematological parameters

The findings of the present study illustrated significant differences in haematological parameters of rainbow trout fed diets supplemented with different levels of NA (Table 1). The highest RBC count was obtained in fish fed the diet supplemented with 50.00 mg kg^−1^ NA, which was significantly higher than that in fish fed with the basal diet (*p* < 0.05). The highest Hb concentration was also recorded in fish fed the diet supplemented with 50.00 mg kg^−1^ NA, although no significant difference was obtained in the Hb concentration of fish by increasing the dietary NA (*p *> 0.05). Fish fed diet supplemented with 50.00 mg kg^−1^ NA had the highest HCT, which was significantly higher than those in fish fed the basal diet (*p* < 0.05). Although the MCH value significantly increased by increasing the dietary NA (*p *< 0.05), no significant difference was recorded in this index among fish fed diets supplemented with 50.00, 75.00 and 100.00 mg kg^−1^ NA. There was also no significant difference in MCV and MCHC among the experimental treatments (*p* > 0.05).

**TABLE 1:** Haematological parameters of rainbow trout, *Oncorhynchus mykiss*, fed diets supplemented with different levels of nanoliposome‐coated astaxanthin for 60 days (mean ± SD).

	Dietary nanoliposome‐coated astaxanthin (mg kg^−1^)				
Variable	0.00 (control)	25.00	50.00	75.00	100.00
RBC **(×10^6^ mm^−3^)**	1.49 ± 0.02^a^	1.63 ± 0.05^b^	1.64 ± 0.01^b^	1.61 ± 0.02^b^	1.56 ± 0.04^ab^
**Hb (g dL^−1^)**	7.13 ± 0.09^a^	7.97 ± 0.20^b^	8.73 ± 0.24^c^	8.60 ± 0.17^c^	8.57 ± 0.09^c^
HCT **(%)**	37.67 ± 0.67^a^	42.67 ± 0.88^bc^	46.67 ± 0.88^c^	45.33 ± 2.33^c^	41.00 ± 0.58^ab^
**MCV (fL)**	252.35 ± 6.27	261.16 ± 9.71	284.08 ± 7.29	282.41 ± 16.37	263.52 ± 2.93
MCH **(pg)**	47.77 ± 0.29^a^	48.69 ± 0.51^a^	53.17 ± 1.83^b^	53.55 ± 1.39^b^	55.10 ± 1.54^b^
**MCHC (mg dL^−1^)**	18.96 ± 0.53	18.70 ± 0.71	18.72 ± 0.42	19.10 ± 1.27	20.90 ± 0.42
WBC **(×10^3^** **mm^−3^)**	3.97 ± 0.09^ab^	4.25 ± 0.07^bc^	5.54 ± 0.21^d^	4.75 ± 0.23^c^	3.62 ± 0.25^a^
**Lymphocytes (%)**	73.00 ± 0.58^b^	72.00 ± 0.58^b^	70.67 ± 0.33^ab^	67.67 ± 0.33^a^	73.67 ± 2.40^b^
**Neutrophils (%)**	21.00 ± 0.58^a^	23.33 ± 0.33^ab^	24.00 ± 0.58^b^	27.33 ± 1.20^c^	21.67 ± 0.88^ab^
**Monocytes (%)**	3.33 ± 0.33	2.67 ± 0.33	3.33 ± 0.33	3.33 ± 0.33	3.67 ± 0.58
**Eosinophils (%)**	1.67 ± 0.67	1.33 ± 0.33	1.33 ± 0.33	1.00 ± 0.00	1.00 ± 0.00

*Note*: Values are shown as mean ± SEM of three replicate tanks (*n* = 3) with three fish per tank. Means in each row with different superscripts have significant differences by Duncan's multiple range test at *p *< 0.05.

Abbreviations: Hb, haemoglobin; HCT, haematocrit; MCH, mean corpuscular haemoglobin; MCHC, mean corpuscular haemoglobin concentration; MCV, mean corpuscular volume; RBC, red blood cell; WBC, white blood cell.

The WBC count was gradually increased by increasing the dietary NA and reached the peak of 5.54 ± 0.21 × 10^3 ^mm^−3^ in fish fed with 50.00 mg kg^−1^ NA. However, the lowest and the highest percentages of lymphocytes and neutrophils were obtained in fish fed with 50.00 mg kg^−1^ NA diet compared to those fed the basal diet (*p *< 0.05). No significant difference was seen in monocyte and eosinophil percentages among the treatments (*p *> 0.05).

### 3.2 Serum biochemical parameters

The alteration in serum biochemical indices of rainbow trout‐fed diets supplemented with different levels of NA is presented in Table 2. Fish receiving the diet supplemented with 50.00 mg kg^−1^ NA had the highest SOD, CAT and GPX activities than those receiving the basal diet (*p *< 0.05), whereas further addition of NA exhibited no significant increase in their activities (*p* > 0.05). Conversely, fish receiving the diet supplemented with 50.00 mg kg^−1^ NA revealed the lowest values of TC, AST and ALT in their sera, which were significantly lower than those receiving the basal diet (*p *< 0.05). A significant decreasing trend was also recorded in MDA value by increasing dietary NA to the minimum of 61.00 ± 0.058 µmol L^−1^ in fish receiving 100.00 mg NA.

**TABLE 2:** Serum antioxidant and biochemical indices of rainbow trout, *Oncorhynchus mykiss*, fed diets supplemented with different levels of nanoliposome‐coated astaxanthin for 60 days (mean ± SD).

	Dietary nanoliposome‐coated astaxanthin (mg kg^−1^)				
Variable	0.00 (control)	25.00	50.00	75.00	100.00
Antioxidant indices					
SOD (IU L^−1^)	62.00 ± 1.73^a^	63.33 ± 3.48^a^	70.67 ± 0.88^b^	64.33 ± 1.33^ab^	64.00 ± 1.73^ab^
CAT (IU L^−1^)	87.33 ± 2.73^a^	98.67 ± 5.04^b^	113.33 ± 4.81^c^	109.33 ± 0.88^bc^	106.67 ± 1.20^bc^
GPX (IU L^−1^)	163.67 ± 1.86^a^	184.33 ± 2.73^b^	200.67 ± 3.48^c^	186.0 ± 2.08^b^	183.33 ± 1.20^b^
Biochemical indices					
MDA (µmol L^−1^)	83.00 ± 1.15^c^	78.67 ± 1.45^c^	72.00 ± 1.53^b^	65.33 ± 2.33^a^	61.00 ± .058^a^
TC (mg dL^−1^)	168.78 ± 1.06^c^	157.86 ± 1.36^b^	147.68 ± 1.67^a^	157.58 ± 2.02^b^	164.31 ± 2.56^c^
AST (IU L^−1^)	293.29 ± 16.37^c^	261.45 ± 13.72^b^	223.64 ± 11.20^a^	239.92 ± 13.70^a^	234.18 ± 12.29^a^
ALT (IU L^−1^)	31.04 ± 0.38^c^	27.82 ± 1.54^b^	23.58 ± 0.26^a^	26.38 ± 0.03^b^	28.70 ± 0.80^bc^

*Note*: ^a^Values are shown as mean ± SEM of three replicate tanks (*n* = 3) with three fish per tank. Means in each row with different superscripts have significant differences by Duncan's multiple range test at *p *< 0.05.

Abbreviations: ALT, alanine aminotransferase; AST, aspartate aminotransferase; CAT, catalase; GPX, glutathione peroxidase; MDA, malondialdehyde; SOD, superoxide dismutase; TC, total cholesterol.

**FIGURE 1:**
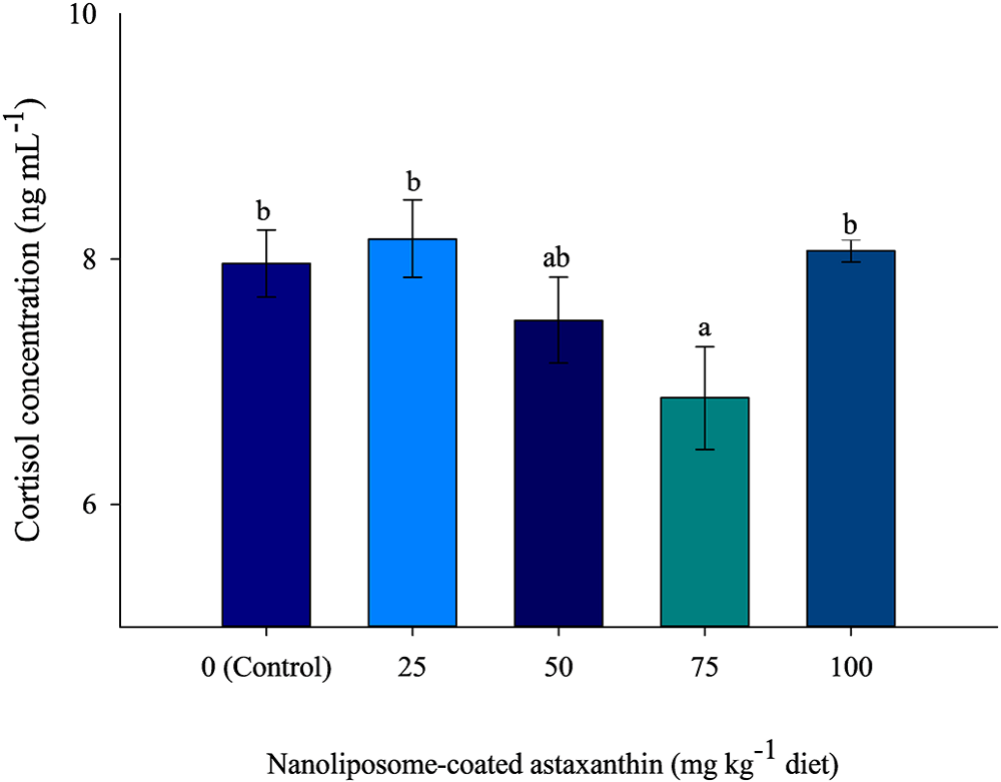
Serum cortisol concentration (ng mL^−1^) of rainbow trout, *Oncorhynchus mykiss*, fed diets supplemented with different levels of nanoliposome‐coated astaxanthin for 60 days. Error bars represent ± standard error of the mean (SEM) of three replicate tanks (*n* = 3) with three fish per tank. Means with different scripts have significant differences by Duncan's multiple range test at *p *< 0.05.

Supplementation of 75.00 mg kg^−1^ NA produced significantly lower serum cortisol than the control group (*p* < 0.05), although no significant difference was recorded in the cortisol level of fish fed diet supplemented with 50.00 and 75.00 NA (*p *> 0.05; Figure 1).

### 3.3 Serum immunological parameters

Serum Lys and ACH50 activities were both significantly stimulated in fish‐fed diets supplemented with NA compared to control fish (*p *< 0.05), although a decreasing trend was recorded by increasing the dietary NA supplementation from 50.00 to 100.00 mg kg^−1^ (Table 3). Serum Ig content significantly increased with increasing dietary NA supplementation up to 75.00 mg kg^−1^ and thereafter decreased (*p *< 0.05).

**TABLE 3:** Serum immunological indices of rainbow trout, *Oncorhynchus mykiss*, fed diets supplemented with different levels of nanoliposome‐coated astaxanthin for 60 days (mean ± SD).

	Dietary nanoliposome‐coated astaxanthin (mg kg^−1^)				
Variable	0.00 (control)	25.00	50.00	75.00	100.00
**Lys (U mL^−1^)**	21.27 ± 0.34^a^	37.09 ± 0.31^d^	43.17 ± 1.46^e^	33.85 ± 1.84^c^	26.36 ± 0.49^b^
**ACH50 (U mL^−1^)**	223.67 ± 4.48^a^	250.67 ± 5.04^c^	293.33 ± 2.03^e^	267.00 ± 2.08^d^	236.00 ± 2.31^b^
**Ig (mg dL^−1^)**	46.00 ± 2.00^a^	51.00 ± 3.61^a^	63.67 ± 3.84^b^	75.67 ± 1.20^c^	53.33 ± 0.67^a^

*Note*: Values are shown as mean ± SEM of three replicate tanks (*n* = 3) with three fish per tank. Means in each row with different superscripts have significant differences by Duncan's multiple range test at *p *< 0.05.

Abbreviations: ACH50, serum alternative complementary activity; Ig, immunoglobulin M; Lys, lysozyme activity.

## 4 DISCUSSION

Nanotechnology is a future‐oriented scientific field that has already impacted many other sectors, such as medicine, cosmetics, energy and food (Kannan et al., 2023; Munekata et al., 2021). In aquaculture, nanoparticle technology has a broad spectrum of applications from pond water treatment and aquatic disease detection to the efficient delivery of cultural inputs, including medicines, vaccines and nutrients, to improve the efficiency and sustainability of the aquaculture industry (Aliko et al., 2024; Gabriel et al., 2022; Jeyavani et al., 2022; Khalil et al., 2022; Paulpandian et al., 2023). However, the application of nanotechnology in aquaculture sector is faced with some limitations, including the cost‐effectiveness of available resources and the practical risks for the application of nanotechnology in aquaculture practices, which should be concerned before industrialization (Munekata et al., 2021). Besides, there is limited information about the impact of nano‐encapsulated natural compounds on the haematology and immunity of aquatic species (Nasr‐Eldahan et al., 2021).

Haematological properties provide some valuable information about the health status and metabolism of several fish species (Burgos‐Aceves et al., 2019; Fazio, Faggio, et al., 2013; Fazio, Marafioti, et al., 2013; Shiry et al., 2023). Few attempts have been made through the years to consider the effects of dietary astaxanthin on the haematological parameters of fish (Lim et al., 2019). Previous investigations expressed the ineffectiveness of dietary supplemented astaxanthin on erythrocytic parameters of Atlantic salmon, *Salmo salar* (Christiansen et al., 1995) and large yellow croaker, *Pseudosciaena crocea* (Li, Wu, et al., 2014). Nonetheless, rainbow trout fed diets supplemented with NA in the present study exhibited higher RBC, Hb and HCT values compared to those fed the basal diet, which indicated that NA increases the function of RBC. In line with these findings, the increase of Hb, HCT and RBC through dietary astaxanthin supplementation has been reported in Asian seabass, *Lates calcarifer* (Lim et al., 2019) and common carp, *Cyprinus carpio* (Jagruthi et al., 2014). These contrasting responses might be attributed to the fish size variation, physiological stage, feeding duration, pigment source, supplementation dose and species‐specific characteristics. Li, Yu et al. (2014) reported that the low value of haemoglobin concentration could be attributed to the peroxidation damage in the cytomembrane of erythrocytes. This condition could force the haematopoietic tissue to stop the proliferation of blood cells, which, in turn, lead to anaemia (Friedman et al., 2004; Xue et al., 2017).

It has been proven that the immune function of fish is closely related to environmental changes in the quality and nutrient structure of food (Mendivil, 2021). Findings of the present study illustrated that an increase in WBC count was correlated with increasing NA in fish diet to up to 50.00 mg NA kg^−1^ diet, reinforcing the notion of much intensive phagocytic activity in supplemented fish. Astaxanthin can span full membrane bilayers and protect against ROS, setting the conditions for optimal WBC production (Foo et al., 2017; Kattappagari et al., 2015).

The potent antioxidant properties of astaxanthin indicate its superior efficacy in stimulating the immune system and function in organs associated with haematopoiesis (Lim et al., 2019). In the present study, rainbow trout fed a diet containing 50 mg kg^−1^ NA revealed the highest neutrophil and lymphocyte percentages. Others have reported that astaxanthin can stimulate the proliferation of neutrophils under in vitro conditions (Macedo et al., 2010). These findings show that astaxanthin, after coating with nanoliposome, has the potential to promote the phagocytic activity of fish immunocompetent cells, probably by reducing the deleterious effects caused by ROS in lipids and proteins of neutrophils and tissues underlying lesions by quenching the exacerbated production of oxidant species (Macedo et al., 2010; Stegenga et al., 2008). It is worth noting that the numbers of leukocytes along with the relative percentage of each type of WBC were within the reference range declared for the health condition of rainbow trout (Nabi et al., 2022; Rozas‐Serri et al., 2022).

Under normal conditions in biological systems, oxygen free radicals are continuously produced and scavenged as a consequence of adenosine triphosphate production, whereas their generation is counterbalanced by antioxidant molecules (Magara et al., 2022). Cells contain a large number of antioxidants to overcome oxidative stress, among which SOD, CAT and GPX are the most enzymatic antioxidants necessary for life in all oxygen‐metabolizing systems (Vélez‐Alavez et al., 2015). Although SOD converts superoxide radicals into hydrogen peroxide and molecular oxygen, CAT and GPX convert the generated hydrogen peroxide into water and oxygen (Cecerska‐Heryć et al., 2021). Various approaches, such as the administration of synthetic antioxidants, have been practiced to enhance the antioxidant defences of fish (Hoseinifar et al., 2021). In the present study, the serum levels of SOD, CAT and GPX were significantly increased by adding the proper amount of NA. These findings confirmed the previous reports that astaxanthin improves the synthesis of enzymatic antioxidants (Li et al., 2018, 2019; Wang et al., 2018; Wu et al., 2017), suggesting that increased antioxidant enzyme activity may be beneficial in suppressing oxidative stress (Yin et al., 2021). However, there are differences in how astaxanthin affects antioxidant enzyme activities. Li, Wu et al. (2014) reported decreased activity of SOD, CAT and GPX in the serum of yellow croaker, *P. crocea*, with increasing dietary astaxanthin supplementation. High levels of astaxanthin inclusion in the diet of post‐smolt Atlantic salmon, *S. salar*, reduced the need for endogenous antioxidant enzymes in plasma or body tissues (Lygren et al., 1999). This difference might be related to the use of astaxanthin in the nanoscale, leading to its higher stimulatory effects, especially on the synthesis of WBCs and other antioxidant enzyme production tissues. This suggestion is further supported by the results of the current study, where the WBC count of rainbow trout was enhanced by increasing the dietary NA. However, an accurate statement needs further research in this field.

The level of MDA, an end‐product of lipid peroxidation, was decreased in the present study with an increase in NA in fish diet, which clearly illustrated the protective effect of astaxanthin in the nanoscale against the oxidative stress of rainbow trout, either by its stimulating effects on the synthesis of antioxidant enzymes or free radical scavenging properties of dietary astaxanthin itself that effectively neutralize oxygen free radicals before they cause the induction of oxidative stress (Chen et al., 2020; Li, Wu, et al., 2014; Zhu et al., 2020). The aminotransferase enzymes, including AST and ALT, are a group of metabolic proteins that are widely used as diagnostic biomarkers to evaluate the liver function of fish (Mehrgan et al., 2022). In the present study, dietary administration of NA attenuated the serum levels of AST and ALT, implying the positive effect of astaxanthin on the nanoscale to protect membrane phospholipids of hepatocytes against peroxidation (Zhu et al., 2020).

Under intensive aquaculture activities and due to the high density of farming, fish are usually exposed to a variety of stressful conditions, such as pathogen invasion, water quality deterioration, handling and confinement (Khanjani et al., 2022). The response to stress in fish is characterized by various physiological responses that lead to neuroendocrine activation and a subsequent cascade of metabolic and physiological changes (Petrovici et al., 2020; Plhalova et al., 2018, 2020; Sadoul & Geffroy, 2019). Cortisol is the main glucocorticoid secreted by the interrenal tissue of teleost fish that mediates the restriction of energy to restore homeostatic regulation (Sadoul & Vijayan, 2016). However, there is little information about the effect of astaxanthin on serum cortisol levels in fish. Zhu et al. (2020) showed that the pretreatment of the Northern snakehead, *Channa argus*, with astaxanthin can mitigate an increase in serum levels of cortisol. A reduced cortisol and glucose level were also reported by feeding of Asian seabass with different concentrations of astaxanthin (Lim et al., 2019). In the current study, fish fed with 75 mg kg^−1^ NA showed significantly lower cortisol levels; these results suggest that NA exhibited an anti‐stress effect in rainbow trout by mitigating the amplitude of elevated cortisol, probably through the inhibition of adrenocorticotropic hormone secretion (Haddad et al., 2013).

There is almost no doubt that a successful aquaculture activity would require maintaining the fish immune system at optimal conditions (Castro & Tafalla, 2015). The innate immune system is considered the first line defence of fish to prevent adherence and colonization of a broad spectrum of pathogen agents (Guo & Dixon, 2021). Among different components of innate immunity, lysozyme has been known as a natural cathionic enzyme found in a wide variety of fish fluid tissues that cleave a glycosidic linkage between *N*‐acetyl muramic acid and *N*‐acetyl glucosamine in the peptidoglycan of bacterial cell walls to increase the opportunity of pathogen phagocytosis in conjunction with complement system (Morrison, 2021). There is a dearth of information about the effect of any carotenoid on the immune responses of fish. In the present study, the level of Lys and ACH50 was significantly enhanced with all levels of NA compared with the control group, implying the positive impacts of dietary astaxanthin in nanoscale on the non‐specific defence of rainbow trout as reported in different fish species, for example large yellow croaker, *P. crocea* (Li, Wu, et al., 2014), yellow catfish, *Pelteobagrus fulvidraco* (Liu et al., 2016), Asian seabass (Li et al., 2019), Northern snakehead (Li et al., 2019) and loach, *Paramisgurnus dabryanus* (Chen et al., 2020), after feeding with different concentrations of bulk astaxanthin. This xanthophyll carotenoid has been approved as a vitamin A precursor and singlet oxygen quencher, reflecting its potential to activate the immune system through both antioxidant and retinoid pathways (Guillou et al., 1989; Kobayashi & Sakamoto, 1999).

Immunoglobulins are a class of heterodimeric glycoprotein proteins secreted by B lymphocytes that act as a critical part of immune responses by recognizing particular pathogens and simplifying their destruction (Shiry et al., 2023; Yu et al., 2020). Although astaxanthin has various antioxidant activities, its immunomodulatory potential and mechanisms have remained unknown. Thompson et al. (1995) reported a negligible effect of astaxanthin on the total Ig of rainbow trout. This is contrary to the findings of Amar et al. (2000), who reported that serum total Ig levels were significantly increased in rainbow trout fed β‐carotene. An obvious increase in Ig was also reported in Asian seabass and Northern snakehead after feeding with different concentrations of bulk astaxanthin (Li et al., 2019; Lim et al., 2019). Obtained data in the present study also illustrated that dietary NA induced the synthesis of total Ig in rainbow trout, suggesting its possible immunoregulatory mechanism in the proliferation and activation of fish B lymphocytes, the most prominent regulators of immunoglobulin synthesis (Ashfaq et al., 2019). The stimulatory effect of astaxanthin on the total Ig content of rainbow trout may be attributed to the downregulatory of cortisol synthesis, which its inhibitory effects have been previously reported on many critical elements of the fish immune system like antibody production, cytokine expression and circulating leukocyte numbers (Ciji & Akhtar, 2021; Ellis et al., 2012; Tort, 2011). However, further research is needed to prove these assumptions.

Although the beneficial effects of astaxanthin on aquatic animals have been extensively studied, its side effects and potential toxicity have not been systematically established or reported (Lim et al., 2018). Experimental evidence from feeding trials shows that the administration of bulk astaxanthin up to 908 mg kg^−1^ diet has no adverse impact on salmonids and ornamental fish (The European Food Safety Authority [EFSA], 2014). However, feeding of rainbow trout with diets supplemented with more than 50 mg kg^−1^ NA in the present study led to a decrease in the serum activity of lysozyme, ACH50 and total Ig contents, indicating that the innate immune response of rainbow trout has been reached to the optimum level with a certain level of NA. This can be due to the nature of liposome nanoparticles, which provide different behaviours in coated astaxanthin compared to the bulk form.

## 5 CONCLUSION

Findings of the current investigation illustrated that NA thrives the haemocyte proliferation, increases haemoglobin synthesis, improves the antioxidant capacity and enhances the immune responses of rainbow trout to enable efficient defence procedures against unfavourable or stressful conditions, such as disease outbreaks, hypoxic condition, thermal stress and osmotic fluctuations, which usually happen in the intensive culture of rainbow trout. These benefits of astaxanthin supplementation of astaxanthin in the nanoform could suggest an alternative strategy for more appropriate use of this pigment and similar compounds to deal with disease and other stressful factors in fish reservoirs.

## AUTHOR CONTRIBUTIONS

*Methodology; investigation; data collection*: Mojdeh Besharat. *Supervision; conceptualization; project administration; revising the original draft*: Houman Rajabi Islami. *Supervision; resources; revising the original draft*: Mehdi Soltani. *Data curation; formal analysis; Writing the original draft*: Seyed Abdolmajid Mousavi.

## CONFLICT OF INTEREST STATEMENT

The authors declare that they have no known financial conflicts of interest or personal relationships that could have appeared to influence the work reported in this paper.

## FUNDING INFORMATION

This study was financially supported by the Islamic Azad University, Science and Research, Tehran, for study designation, samples collection, and data interpretation.

### ETHICS STATEMENT

All the experimental protocols involving the fish in the present study were carried out according to the Iranian animal ethics framework. In addition, the proposal and the entire experimental protocol were reviewed and approved by the ethics committee for conducting medical research in Iran (Approval ID: IR.IAU.SRB.REC.1399.108).

### PEER REVIEW

The peer review history for this article is available at https://publons.com/publon/10.1002/vms3.1461.

## Data Availability

The data that support the findings of this study are available on request from the corresponding authors.

## References

[vms31461-bib-0001] Ahmadpanah, K. , Soltani, M. , Rajabi Islami, H. , & Shamsaie, M. (2019). Effects of nonylphenol on hematological parameters and immune responses in immature rainbow trout (*Oncorhynchus mykiss*). Marine and Freshwater Behaviour and Physiology, 52(4), 151–165. 10.1080/10236244.2019.1661779

[vms31461-bib-0002] Aliko, V. , Vasjari, L. , Ibrahimi, E. , Impellitteri, F. , Karaj, A. , Gjonaj, G. , Piccione, G. , Arfuso, F. , Faggio, C. , & Istifli, E. S. (2024). “From shadows to shores”‐quantitative analysis of CuO nanoparticle‐induced apoptosis and DNA damage in fish erythrocytes: A multimodal approach combining experimental, image‐based quantification, docking and molecular dynamics. Science of the Total Environment, 906, 167698. 10.1016/j.scitotenv.2023.167698 37832669

[vms31461-bib-0003] Amar, E. C. , Kiron, V. , Satoh, S. , Okamoto, N. , & Watanabe, T. (2000). Effects of dietary β‐carotene on the immune response of rainbow trout *Oncorhynchus mykiss* . Fisheries Science, 66, 1068–1075. 10.1046/J.1444-2906.2000.00170.X

[vms31461-bib-0004] Arab, N. , & Rajabi Islami, H. (2015). Effects of dietary ascorbic acid on growth performance, body composition, and some immunological parameters of Caspian brown trout, *Salmo trutta caspius* . Journal of the World Aquaculture Society, 46(5), 505–518. 10.1111/jwas.12215

[vms31461-bib-0005] Ashfaq, H. , Soliman, H. , Saleh, M. , & El‐Matbouli, M. (2019). CD4: A vital player in the teleost fish immune system. Veterinary Research, 50(1), 1. 10.1186/s13567-018-0620-0 30616664 PMC6323851

[vms31461-bib-0006] Basiony, M. , Ouyang, L. , Wang, D. , Yu, J. , Zhou, L. , Zhu, M. , Wang, X. , Feng, J. , Dai, J. , Shen, Y. , Zhang, C. , Hua, Q. , Yang, X. , & Zhang, L. (2022). Optimization of microbial cell factories for astaxanthin production: Biosynthesis and regulations, engineering strategies and fermentation optimization strategies. Synthetic and Systems Biotechnology, 7(2), 689–704. 10.1016/j.synbio.2022.01.002 35261927 PMC8866108

[vms31461-bib-0007] Bassijeh, A. , Ansari, S. , & Hosseini, S. M. H. (2020). Astaxanthin encapsulation in multilayer emulsions stabilized by complex coacervates of whey protein isolate and Persian gum and its use as a natural colorant in a model beverage. Food Research International, 137, 109689. 10.1016/j.foodres.2020.109689 33233264

[vms31461-bib-0008] Besharat, M. , Rajabi Islami, H. , Soltani, M. , & Abdolmajid Mousavi, S. (2021). Effect of different levels of nanoliposome‐coated astasxanthin on growth performance, body proximate composition, liver enzyme activity and pigmentation of rainbow trout (*Oncorhynchus mykiss*). Aquaculture Research, 52(10), 5069–5077. 10.1111/are.15378

[vms31461-bib-0009] Burgos‐Aceves, M. A. , Lionetti, L. , & Faggio, C. (2019). Multidisciplinary haematology as prognostic device in environmental and xenobiotic stress‐induced response in fish. Science of the Total Environment, 670, 1170–1183. 10.1016/j.scitotenv.2019.03.275 31018433

[vms31461-bib-0010] Capelli, B. , Bagchi, D. , & Cysewski, G. R. (2013). Synthetic astaxanthin is significantly inferior to algal‐based astaxanthin as an antioxidant and may not be suitable as a human nutraceutical supplement. Nutrafoods, 12(4), 145–152. 10.1007/s13749-013-0051-5

[vms31461-bib-0011] Castro, R. , & Tafalla, C. (2015). Overview of fish immunity. In B. H. Beck , & E. Peatman (Eds.), Mucosal health in aquaculture (pp. 3–54). Academic Press.

[vms31461-bib-0012] Cecerska‐Heryć, E. , Surowska, O. , Heryć, R. , Serwin, N. , Napiontek‐Balińska, S. , & Dołęgowska, B. (2021). Are antioxidant enzymes essential markers in the diagnosis and monitoring of cancer patients—A review. Clinical Biochemistry, 93, 1–8. 10.1016/j.clinbiochem.2021.03.008 33773993

[vms31461-bib-0013] Chariou, P. L. , Ortega‐Rivera, O. A. , & Steinmetz, N. F. (2020). Nanocarriers for the delivery of medical, veterinary, and agricultural active ingredients. ACS Nano, 14(3), 2678–2701. 10.1021/acsnano.0c00173 32125825 PMC8085836

[vms31461-bib-0014] Chen, X.‐M. , Gao, C.‐S. , Du, X.‐Y. , Yao, J.‐M. , He, F.‐F. , Niu, X.‐T. , Wang, G.‐Q. , & Zhang, D.‐M. (2020). Effects of dietary astaxanthin on the growth, innate immunity and antioxidant defence system of *Paramisgurnus dabryanus* . Aquaculture Nutrition, 26(5), 1453–1462. 10.1111/anu.13093

[vms31461-bib-0015] Christiansen, R. , Glette, J. , Lie, Ø. , Torrissen, O. J. , & Waagbø, R. (1995). Antioxidant status and immunity in Atlantic salmon, *Salmo salar* L., fed semi‐purified diets with and without astaxanthin supplementation. Journal of Fish Diseases, 18(4), 317–328. 10.1111/j.1365-2761.1995.tb00308.x

[vms31461-bib-0016] Ciji, A. , & Akhtar, M. S. (2021). Stress management in aquaculture: A review of dietary interventions. Reviews in Aquaculture, 13(4), 2190–2247. 10.1111/raq.12565

[vms31461-bib-0017] Dethlefsen, M. W. , Hjermitslev, N. H. , Frosch, S. , & Nielsen, M. E. (2016). Effect of storage on oxidative quality and stability of extruded astaxanthin‐coated fish feed pellets. Animal Feed Science and Technology, 221, 157–166. 10.1016/j.anifeedsci.2016.08.007

[vms31461-bib-0018] Dose, J. , Matsugo, S. , Yokokawa, H. , Koshida, Y. , Okazaki, S. , Seidel, U. , Eggersdorfer, M. , Rimbach, G. , & Esatbeyoglu, T. (2016). Free radical scavenging and cellular antioxidant properties of astaxanthin. International Journal of Molecular Sciences, 17(1). 10.3390/ijms17010103 PMC4730345 26784174

[vms31461-bib-0019] Dufossé, L. (2008). Pigments from microalgae and microorganisms: Sources of food colorants. In C. Socaciu , & E. Z. Sikorski (Eds.), Food colorants, chemical and functional properties (pp. 399–426). CRC Press.

[vms31461-bib-0021] Ellis, A. E. (1990). Lysozyme assays. In J. S. Stolen , T. C. Fletcher , D. P. Anderson , B. S. Roberson , & W. B. Van Muiswinkel (Eds.), Techniques in fish immunology (pp. 101–103). SOS Publications.

[vms31461-bib-0022] Ellis, T. , Yildiz, H. Y. , López‐Olmeda, J. , Spedicato, M. T. , Tort, L. , Øverli, Ø. , & Martins, C. I. M. (2012). Cortisol and finfish welfare. Fish Physiology and Biochemistry, 38(1), 163–188. 10.1007/s10695-011-9568-y 22113503

[vms31461-bib-0023] Fang, N. , Wang, C. , Liu, X. , Zhao, X. , Liu, Y. , Liu, X. , Du, Y. , Zhang, Z. , & Zhang, H. (2019). De novo synthesis of astaxanthin: From organisms to genes. Trends in Food Science & Technology, 92, 162–171. 10.1016/j.tifs.2019.08.016

[vms31461-bib-0024] Farahnak Roudsari, S. , Rajabi Islami, H. , Mousavi, S. A. , & Shamsaie Mehrgan, M. (2021). Folic acid‐coated nanochitosan ameliorated the growth performance, hematological parameters, antioxidant status, and immune responses of rainbow trout (*Oncorhynchus mykiss*). Frontiers in Veterinary Science, 8, 647722. 10.3389/fvets.2021.64772 34212018 PMC8241213

[vms31461-bib-0025] Fazio, F. , Faggio, C. , Marafioti, S. , Torre, A. , Sanfilippo, M. , & Piccione, G. (2013). Effect of water quality on hematological and biochemical parameters of *Gobius niger* caught in Faro lake (Sicily). IFRO, 12(1), 219–231. http://jifro.ir/article‐1‐884‐en.html

[vms31461-bib-0026] Fazio, F. , Marafioti, S. , Torre, A. , Sanfilippo, M. , Panzera, M. , & Faggio, C. (2013). Haematological and serum protein profiles of *Mugil cephalus*: Effect of two different habitats. Ichthyological Research, 60(1), 36–42. 10.1007/s10228-012-0303-1

[vms31461-bib-0027] Foo, S. C. , Yusoff, F. M. , Ismail, M. , Basri, M. , Yau, S. K. , Khong, N. M. H. , Chan, K. W. , & Ebrahimi, M. (2017). Antioxidant capacities of fucoxanthin‐producing algae as influenced by their carotenoid and phenolic contents. Journal of Biotechnology, 241, 175–183. 10.1016/j.jbiotec.2016.11.026 27914891

[vms31461-bib-0028] Friedman, J. S. , Lopez, M. F. , Fleming, M. D. , Rivera, A. , Martin, F. M. , Welsh, M. L. , Boyd, A. , Doctrow, S. R. , & Burakoff, S. J. (2004). SOD2‐deficiency anemia: Protein oxidation and altered protein expression reveal targets of damage, stress response, and antioxidant responsiveness. Blood, 104(8), 2565–2573. 10.1182/blood-2003-11-3858 15205258

[vms31461-bib-0029] Gabriel, N. N. , Habte‐Tsion, H.‐M. , & Haulofu, M. (2022). Perspectives of nanotechnology in aquaculture: Fish nutrition, disease, and water treatment. In A. Krishnan , B. Ravindran , B. Balasubramanian , H. C. Swart , S. J. Panchu , & R. Prasad (Eds.), Emerging nanomaterials for advanced technologies (pp. 463–485). Springer International Publishing.

[vms31461-bib-0030] Guerin, M. , Huntley, M. E. , & Olaizola, M. (2003). *Haematococcus* astaxanthin: Applications for human health and nutrition. Trends in Biotechnology, 21(5), 210–216. 10.1016/S0167-7799(03)00078-7 12727382

[vms31461-bib-0031] Guillou, A. , Choubert, G. , Storebakken, T. , De La Noüet, J. , & Kaushik, S. (1989). Bioconversion pathway of astaxanthin into retinol2 in mature rainbow trout (*Salmo gairdneri rich*.). Comparative Biochemistry and Physiology Part B: Comparative Biochemistry, 94(3), 481–485. 10.1016/0305-0491(89)90185-5

[vms31461-bib-0032] Guo, H. , & Dixon, B. (2021). Understanding acute stress‐mediated immunity in teleost fish. Fish and Shellfish Immunology Reports, 2, 100010. 10.1016/j.fsirep.2021.100010 36420509 PMC9680050

[vms31461-bib-0033] Haddad, N. F. , Teodoro, A. J. , Leite De Oliveira, F. , Soares, N. , De Mattos, R. M. , Hecht, F. , Dezonne, R. S. , Vairo, L. , Goldenberg, R. , Gomes, F. C. A. , De Carvalho, D. P. , Gadelha, M. R. , Nasciutti, L. E. , & Miranda‐Alves, L. (2013). Lycopene and beta‐carotene induce growth inhibition and proapoptotic effects on ACTH‐secreting pituitary adenoma cells. PLoS One, 8(5), e62773. 10.1371/journal.pone.0062773 23667519 PMC3647049

[vms31461-bib-0034] Hoseinifar, S. H. , Yousefi, S. , Van Doan, H. , Ashouri, G. , Gioacchini, G. , Maradonna, F. , & Carnevali, O. (2021). Oxidative stress and antioxidant defense in fish: The implications of probiotic, prebiotic, and synbiotics. Reviews in Fisheries Science & Aquaculture, 29(2), 198–217. 10.1080/23308249.2020.1795616

[vms31461-bib-0035] Hu, I. C. (2019). Production of potential coproducts from microalgae. In A. Pandey , J.‐S. Chang , C. R. Soccol , D.‐J. Lee , & Y. Chisti (Eds.), Biofuels from algae (2nd ed., pp. 345–358). Elsevier.

[vms31461-bib-0036] Jagruthi, C. , Yogeshwari, G. , Anbazahan, S. M. , Shanthi Mari, L. S. , Arockiaraj, J. , Mariappan, P. , Sudhakar, G. R. , Balasundaram, C. , & Harikrishnan, R. (2014). Effect of dietary astaxanthin against *Aeromonas hydrophila* infection in common carp, *Cyprinus carpio* . Fish & Shellfish Immunology, 41(2), 674–680. 10.1016/j.fsi.2014.10.010 25462460

[vms31461-bib-0037] Jamalzad Falah, F. , Rajabi Islami, H. , & Shamsaie Mehrgan, M. (2020). Dietary folic acid improved growth performance, immuno‐physiological response and antioxidant status of fingerling Siberian sturgeon, *Acipenser baerii* (Brandt 1896). Aquaculture Reports, 17, 100391. 10.1016/j.aqrep.2020.100391

[vms31461-bib-0038] Jeyavani, J. , Sibiya, A. , Sivakamavalli, J. , Divya, M. , Preetham, E. , Vaseeharan, B. , & Faggio, C. (2022). Phytotherapy and combined nanoformulations as a promising disease management in aquaculture: A review. Aquaculture International, 30(2), 1071–1086. 10.1007/s10499-022-00848-0

[vms31461-bib-0039] Kalinowski, C. T. , Larroquet, L. , Véron, V. , Robaina, L. , Izquierdo, M. S. , Panserat, S. , Kaushik, S. , & Fontagné‐Dicharry, S. (2019). Influence of dietary astaxanthin on the hepatic oxidative stress response caused by episodic hyperoxia in rainbow trout. Antioxidants, 8(12), 626. 10.3390/antiox8120626 31817693 PMC6943655

[vms31461-bib-0040] Kannan, M. , Bojan, N. , Swaminathan, J. , Zicarelli, G. , Hemalatha, D. , Zhang, Y. , Ramesh, M. , & Faggio, C. (2023). Nanopesticides in agricultural pest management and their environmental risks: A review. International Journal of Environmental Science and Technology, 20(9), 10507–10532. 10.1007/s13762-023-04795-y

[vms31461-bib-0041] Kattappagari, K. , Ravi Teja, C. , Kommalapati, R. , Poosarla, C. , Gontu, S. , & Reddy, B. R. (2015). Role of antioxidants in facilitating the body functions: A review. Journal of Orofacial Sciences, 7(2), 71–75. 10.4103/0975-8844.169745

[vms31461-bib-0042] Khalil, H. S. , Maulu, S. , Verdegem, M. , & Abdel‐Tawwab, M. (2022). Embracing nanotechnology for selenium application in aquafeeds. Reviews in Aquaculture, 15(1), 112–129. 10.1111/raq.12705

[vms31461-bib-0043] Khanjani, M. H. , Zahedi, S. , & Mohammadi, A. (2022). Integrated multitrophic aquaculture (IMTA) as an environmentally friendly system for sustainable aquaculture: Functionality, species, and application of biofloc technology (BFT). Environmental Science and Pollution Research, 29, 67513–67531. 10.1007/s11356-022-22371-8 35922597

[vms31461-bib-0044] Khorasani, S. , Danaei, M. , & Mozafari, M. R. (2018). Nanoliposome technology for the food and nutraceutical industries. Trends in Food Science & Technology, 79, 106–115. 10.1016/j.tifs.2018.07.009

[vms31461-bib-0045] Kobayashi, M. , & Sakamoto, Y. (1999). Singlet oxygen quenching ability of astaxanthin esters from the green alga *Haematococcus pluvialis* . Biotechnology Letters, 21(4), 265–269. 10.1023/A:1005445927433

[vms31461-bib-0046] Li, F. , Huang, S. , Lu, X. , Wang, J. , Lin, M. , An, Y. , Wu, S. , & Cai, M. (2018). Effects of dietary supplementation with algal astaxanthin on growth, pigmentation, and antioxidant capacity of the blood parrot (*Cichlasoma citrinellum* × *Cichlasoma synspilum*). Journal of Oceanology and Limnology, 36(5), 1851–1859. 10.1007/s00343-019-7172-7

[vms31461-bib-0047] Li, M.‐Y. , Liu, X.‐Y. , Xia, C.‐G. , Wang, G.‐Q. , & Zhang, D.‐M. (2019). Astaxanthin enhances hematology, antioxidant and immunological parameters, immune‐related gene expression, and disease resistance against in *Channa argus* . Aquaculture International, 27(3), 735–746. 10.1007/s10499-019-00362-w

[vms31461-bib-0048] Li, M. , Wu, W. , Zhou, P. , Xie, F. , Zhou, Q. , & Mai, K. (2014). Comparison effect of dietary astaxanthin and *Haematococcus pluvialis* on growth performance, antioxidant status and immune response of large yellow croaker *Pseudosciaena crocea* . Aquaculture, 434, 227–232. 10.1016/j.aquaculture.2014.08.022

[vms31461-bib-0049] Li, M. , Yu, N. , Qin, J. G. , Li, E. , Du, Z. , & Chen, L. (2014). Effects of ammonia stress, dietary linseed oil and *Edwardsiella ictaluri* challenge on juvenile darkbarbel catfish *Pelteobagrus vachelli* . Fish & Shellfish Immunology, 38(1), 158–165. 10.1016/j.fsi.2014.03.015 24657724

[vms31461-bib-0050] Lim, K. C. , Yusoff, F. M. , Shariff, M. , & Kamarudin, M. S. (2018). Astaxanthin as feed supplement in aquatic animals. Reviews in Aquaculture, 10(3), 738–773. 10.1111/raq.12200

[vms31461-bib-0051] Lim, K. C. , Yusoff, F. M. , Shariff, M. , Kamarudin, M. S. , & Nagao, N. (2019). Dietary supplementation of astaxanthin enhances hemato‐biochemistry and innate immunity of Asian seabass, *Lates calcarifer* (Bloch, 1790). Aquaculture, 512, 734339. 10.1016/j.aquaculture.2019.734339

[vms31461-bib-0052] Lin, K.‐H. , Lin, K.‐C. , Lu, W.‐J. , Thomas, P.‐A. , Jayakumar, T. , & Sheu, J.‐R. (2015). Astaxanthin, a carotenoid, stimulates immune responses by enhancing IFN‐γ and IL‐2 secretion in primary cultured lymphocytes in vitro and ex vivo. International Journal of Molecular Sciences, 17(1), 44. 10.3390/ijms17010044 26729100 PMC4730289

[vms31461-bib-0053] Liu, C. , Tan, Y. , Xu, Y. , Mccleiments, D. J. , & Wang, D. (2019). Formation, characterization, and application of chitosan/pectin‐stabilized multilayer emulsions as astaxanthin delivery systems. International Journal of Biological Macromolecules, 140, 985–997. 10.1016/j.ijbiomac.2019.08.071 31401274

[vms31461-bib-0054] Liu, F. , Shi, H.‐Z. , Guo, Q.‐S. , Yu, Y.‐B. , Wang, A.‐M. , Lv, F. , & Shen, W.‐B. (2016). Effects of astaxanthin and emodin on the growth, stress resistance and disease resistance of yellow catfish (*Pelteobagrus fulvidraco*). Fish & Shellfish Immunology, 51, 125–135. 10.1016/j.fsi.2016.02.020 26899124

[vms31461-bib-0055] Liu, S. , Lian, J. , Xu, Z. , Ning, Y. , Shi, M. , Zhao, Z. , & Zhang, Z. (2022). Chitosan‐coated nanoliposomes for efficient delivery of betanin with enhanced stability and bioavailability. Food Hydrocolloids, 132, 107871. 10.1016/j.foodhyd.2022.107871

[vms31461-bib-0056] Lu, Q. , Li, H. , Zou, Y. , Liu, H. , & Yang, L. (2021). Astaxanthin as a microalgal metabolite for aquaculture: A review on the synthetic mechanisms, production techniques, and practical application. Algal Research, 54, 102178. 10.1016/j.algal.2020.102178

[vms31461-bib-0057] Lygren, B. , Hamre, K. , & Waagbø, R. (1999). Effects of dietary pro‐ and antioxidants on some protective mechanisms and health parameters in Atlantic salmon. Journal of Aquatic Animal Health, 11(3), 211–221. 10.1577/1548-8667(1999)011≤0211:EODPAA≥2.0.CO;2.

[vms31461-bib-0058] Macedo, R. C. , Bolin, A. P. , Marin, D. P. , & Otton, R. (2010). Astaxanthin addition improves human neutrophils function: In vitro study. European Journal of Nutrition, 49(8), 447–457. 10.1007/s00394-010-0103-1 20361333

[vms31461-bib-0059] Magara, G. , Prearo, M. , Vercelli, C. , Barbero, R. , Micera, M. , Botto, A. , Caimi, C. , Caldaroni, B. , Bertea, C. M. , Mannino, G. , Barceló, D. , Renzi, M. , Gasco, L. , Re, G. , Dondo, A. , Elia, A. C. , & Pastorino, P. (2022). Modulation of antioxidant defense in farmed rainbow trout (*Oncorhynchus mykiss*) fed with a diet supplemented by the waste derived from the supercritical fluid extraction of basil (*Ocimum basilicum*). Antioxidants (Basel), 11(2), 415. 10.3390/antiox11020415 35204297 PMC8869336

[vms31461-bib-0060] Martínez‐Delgado, A. A. , Khandual, S. , & Villanueva–Rodríguez, S. J. (2017). Chemical stability of astaxanthin integrated into a food matrix: Effects of food processing and methods for preservation. Food Chemistry, 225, 23–30. 10.1016/j.foodchem.2016.11.092 28193419

[vms31461-bib-0061] Mehrgan, M. S. , Shekarabi, S. P. H. , Azari, A. , Yilmaz, S. , Lückstädt, C. , & Rajabi Islami, H. (2022). Synergistic effects of sodium butyrate and sodium propionate on the growth performance, blood biochemistry, immunity, and immune‐related gene expression of goldfish (*Carassius auratus*). Aquaculture International, 30, 3179–3193. 10.1007/s10499-022-00954-z

[vms31461-bib-0062] Mendivil, C. O. (2021). Dietary fish, fish nutrients, and immune function: A review. Frontiers in Nutrition, 7, 617652. 10.3389/fnut.2020.617652 33553231 PMC7855848

[vms31461-bib-0063] Moazenzadeh, K. , Rajabi Islami, H. , Zamini, A. , & Soltani, M. (2020). Effect of dietary inorganic copper on growth performance and some hematological indices of Siberian sturgeon *Acipenser baerii* juveniles. North American Journal of Aquaculture, 82(2), 200–207. 10.1002/naaq.10145

[vms31461-bib-0064] Mohammadi, M. , & Khara, H. (2015). Effect of different anesthetic agents (clove oil, tricaine methanesulfonate, ketamine, tobacco) on hematological parameters and stress indicators of rainbow trout *Oncorhynchus mykiss*, Walbaum, 1792. Comparative Clinical Pathology, 24(5), 1039–1044. 10.1007/s00580-014-2027-2

[vms31461-bib-0065] Morrison, H. (2021). Lysozyme. In H. Morrison (Ed.), Enzyme active sites and their reaction mechanisms (pp. 121–127). Academic Press.

[vms31461-bib-0066] Mostafavi, Z. S. , Shekarabi, S. P. H. , Mehrgan, M. S. , & Islami, H. R. (2022). Amelioration of growth performance, physio‐metabolic responses, and antioxidant defense system in rainbow trout, *Oncorhynchus mykiss*, using dietary dandelion, *Taraxacum officinale*, flower extract. Aquaculture, 546, 737296. 10.1016/j.aquaculture.2021.737296

[vms31461-bib-0067] Munekata, P. E. S. , Pateiro, M. , Domínguez, R. , Farag, M. A. , Varzakas, T. , & Lorenzo, J. M. (2021). Nanotechnology. In J. M. Lorenzo , P. E. S. Munekata , & F. J. Barba (Eds.), Sustainable production technology in food (pp. 179–202). Academic Press.

[vms31461-bib-0068] Muthuraman, A. , Shaikh, S. A. , Ramesh, M. , & Sikarwar, M. S. (2021). The structure‐activity relationship of marine products for neuroinflammatory disorders. In R. Attaur (Ed.), Studies in natural products chemistry (Vol. 70, pp. 151–194). Elsevier.

[vms31461-bib-0069] Nabi, N. , Ahmed, I. , & Wani, G. B. (2022). Hematological and serum biochemical reference intervals of rainbow trout, *Oncorhynchus mykiss* cultured in Himalayan aquaculture: Morphology, morphometrics and quantification of peripheral blood cells. Saudi Journal of Biological Sciences, 29(4), 2942–2957. 10.1016/j.sjbs.2022.01.019 35531244 PMC9073141

[vms31461-bib-0070] Najafi, Z. , Bildik, F. , Şahin‐Yeşilçubuk, N. , & Altay, F. (2022). Enhancing oxidative stability of encapsulated echium oil by incorporation of saffron extract loaded nanoliposomes into electrospun pullulan‐pea protein isolate‐pectin. Food Hydrocolloids, 129, 107627. 10.1016/j.foodhyd.2022.107627

[vms31461-bib-0071] Nasr‐Eldahan, S. , Nabil‐Adam, A. , Shreadah, M. A. , Maher, A. M. , & El‐Sayed Ali, T. (2021). A review article on nanotechnology in aquaculture sustainability as a novel tool in fish disease control. Aquaculture International, 29(4), 1459–1480. 10.1007/s10499-021-00677-7 33688117 PMC7933385

[vms31461-bib-0072] Niizawa, I. , Espinaco, B. Y. , Zorrilla, S. E. , & Sihufe, G. A. (2021). Astaxanthin production by autotrophic cultivation of *Haematococcus pluvialis*: A success story. In G. A. Ravishankar , & A. R Rao (Eds.), Global perspectives on astaxanthin (pp. 71–89). Academic Press.

[vms31461-bib-0073] Ohkawa, H. , Ohishi, N. , & Yagi, K. (1979). Assay for lipid peroxides in animal tissues by thiobarbituric acid reaction. Analytical Biochemistry, 95(2), 351–358. 10.1016/0003-2697(79)90738-3 36810

[vms31461-bib-0074] Pan, L. , Wang, H. , & Gu, K. (2018). Nanoliposomes as vehicles for astaxanthin: Characterization, in vitro release evaluation and structure. Molecules, 23(11). 10.3390/molecules23112822 PMC6278380 30380797

[vms31461-bib-0075] Pan, L. , Zhang, S. , Gu, K. , & Zhang, N. (2018). Preparation of astaxanthin‐loaded liposomes: Characterization, storage stability and antioxidant activity. CYTA—Journal of Food, 16(1), 607–618. 10.1080/19476337.2018.1437080

[vms31461-bib-0076] Panahi, Y. , Farshbaf, M. , Mohammadhosseini, M. , Mirahadi, M. , Khalilov, R. , Saghfi, S. , & Akbarzadeh, A. (2017). Recent advances on liposomal nanoparticles: synthesis, characterization and biomedical applications. Artificial Cells, Nanomedicine, and Biotechnology, 45(4), 788–799. 10.1080/21691401.2017.1282496 28278586

[vms31461-bib-0077] Paulpandian, P. , Beevi, I. S. , Somanath, B. , Kamatchi, R. K. , Paulraj, B. , & Faggio, C. (2023). Impact of *Camellia sinensis* iron oxide nanoparticle on growth, hemato‐biochemical and antioxidant capacity of blue gourami (*Trichogaster trichopterus*) fingerlings. Biological Trace Element Research, 201(1), 412–424. 10.1007/s12011-022-03145-2 35201568

[vms31461-bib-0078] Petrovici, A. , Strungaru, S.‐A. , Nicoara, M. , Robea, M. A. , Solcan, C. , & Faggio, C. (2020). Toxicity of deltamethrin to zebrafish gonads revealed by cellular biomarkers. Journal of Marine Science and Engineering, 8(2), 73. 10.3390/jmse8020073

[vms31461-bib-0079] Plhalova, L. , Blahova, J. , Divisova, L. , Enevova, V. , Casuscelli Di Tocco, F. , Faggio, C. , Tichy, F. , Vecerek, V. , & Svobodova, Z. (2018). The effects of subchronic exposure to NeemAzal T/S on zebrafish (*Danio rerio*). Chemistry and Ecology, 34(3), 199–210. 10.1080/02757540.2017.1420176

[vms31461-bib-0080] Plhalova, L. , Sehonova, P. , Blahova, J. , Doubkova, V. , Tichy, F. , Faggio, C. , Berankova, P. , & Svobodova, Z. (2020). Evaluation of tramadol hydrochloride toxicity to juvenile zebrafish—Morphological, antioxidant and histological responses. Applied Sciences, 10(7), 2349. 10.3390/app10072349

[vms31461-bib-0081] Reitman, S. , & Frankel, S. (1957). A colorimetric method for the determination of serum glutamic oxalacetic and glutamic pyruvic transaminases. American Journal of Clinical Pathology, 28(1), 56–63. 10.1093/ajcp/28.1.56 13458125

[vms31461-bib-0082] Rozas‐Serri, M. , Correa, R. , Walker‐Vergara, R. , Coñuecar, D. , Barrientos, S. , Leiva, C. , Ildefonso, R. , Senn, C. , & Peña, A. (2022). Reference intervals for blood biomarkers in farmed Atlantic salmon, Coho salmon and rainbow trout in Chile: Promoting a preventive approach in aquamedicine. Biology, 11(7), 1066. 10.3390/biology11071066 36101444 PMC9312075

[vms31461-bib-0083] Sadoul, B. , & Geffroy, B. (2019). Measuring cortisol, the major stress hormone in fishes. Journal of Fish Biology, 94(4), 540–555. 10.1111/jfb.13904 30667059

[vms31461-bib-0084] Sadoul, B. , & Vijayan, M. M. (2016). Stress and growth. In C. B. Schreck , L. Tort , A. P. Farrell , & C. J. Brauner (Eds.), Fish physiology (Vol. 35, pp. 167–205). Academic Press.

[vms31461-bib-0085] Saheli, M. , Rajabi Islami, H. , Mohseni, M. , & Soltani, M. (2021). Effects of dietary vitamin E on growth performance, body composition, antioxidant capacity, and some immune responses in Caspian trout (*Salmo caspius*). Aquaculture Reports, 21, 100857. 10.1016/j.aqrep.2021.100857

[vms31461-bib-0086] Sarabandi, K. , Rafiee, Z. , Khodaei, D. , & Jafari, S. M. (2019). Encapsulation of food ingredients by nanoliposomes. In S. M. Jafari (Ed.), Lipid‐based nanostructures for food encapsulation purposes (pp. 347–404). Academic Press.

[vms31461-bib-0087] Shiry, N. , Alavinia, S. J. , Impellitteri, F. , Alavinia, S. J. , & Faggio, C. (2023). Beyond the surface: Consequences of methyl tert‐butyl ether (MTBE) exposure on oxidative stress, haematology, genotoxicity, and histopathology in rainbow trout. Science of the Total Environment, 900, 165784. 10.1016/j.scitotenv.2023.165784 37499819

[vms31461-bib-0088] Siwicki, A. K. , & Anderson, D. P. (1993). Nonspecific defence mechanisms assay in fish II; Potential killing activity of neutrophils and manocytes, lysozyme activity in serum and organs and total immunoglobulin (Ig) level in serum. In A. K. Siwicki , D. P. Anderson , & J. Waluga (Eds.), Disease Diagnosis and Prevention Methods (pp. 105–111). FAO‐Project GCP/INT/526/JPN.

[vms31461-bib-0089] Stegenga, M. E. , Crabben, S. , Dessing, M. C. , Pater, J. M. , Van Den Pangaart, P. S. , De Vos, A. F. , Tanck, M. W. , Roos, D. , Sauerwein, H. P. , & Van Der Poll, T. (2008). Effect of acute hyperglycaemia and/or hyperinsulinaemia on proinflammatory gene expression, cytokine production and neutrophil function in humans. Diabetic Medicine, 25(2), 157–164. 10.1111/j.1464-5491.2007.02348.x 18290856 PMC2268957

[vms31461-bib-0090] Su, W. , Polyakov, N. E. , Xu, W. , & Su, W. (2021). Preparation of astaxanthin micelles self‐assembled by a mechanochemical method from hydroxypropyl β‐cyclodextrin and glyceryl monostearate with enhanced antioxidant activity. International Journal of Pharmaceutics, 605, 120799. 10.1016/j.ijpharm.2021.120799 34126176

[vms31461-bib-0020] The European Food Safety Authority (EFSA) . (2014). Scientific opinion on the safety and efficacy of astaxanthin (CAROPHYLL^®^ Pink 10% CWS) for salmonids and ornamental fish. EFSA Journal, 1, 1–35. 10.2903/j.efsa.2014.3725

[vms31461-bib-0091] Thompson, I. , Choubert, G. , Houlihan, D. F. , & Secombes, C. J. (1995). The effect of dietary vitamin A and astaxanthin on the immunocompetence of rainbow trout. Aquaculture, 133(2), 91–102. 10.1016/0044-8486(95)00024-V

[vms31461-bib-0092] Tort, L. (2011). Stress and immune modulation in fish. Developmental & Comparative Immunology, 35(12), 1366–1375. 10.1016/j.dci.2011.07.002 21782845

[vms31461-bib-0093] Varma, K. , Jude, S. , Nair, R. V. R. , Varghese, B. A. , Jacob, J. , Amalraj, A. , & Kuttappan, S. (2021). Novel formulation of liposomal lutein using nanofiber weaving (NFW) technology: Antioxidant property and in vitro release studies. Food Hydrocolloids for Health, 1, 100025. 10.1016/j.fhfh.2021.100025

[vms31461-bib-0094] Vélez‐Alavez, M. , De Anda‐Montañez, J. A. , Galván‐Magaña, F. , & Zenteno‐Savín, T. (2015). Comparative study of enzymatic antioxidants in muscle of elasmobranch and teleost fishes. Comparative Biochemistry and Physiology Part A: Molecular & Integrative Physiology, 187, 61–65. 10.1016/j.cbpa.2015.04.014 25952027

[vms31461-bib-0095] Verlhac Trichet, V. , & Amaya, E. (2022). Astaxanthin use as carotenoid source and its benefits in feeds. In D. A. Davis (Ed.), Feed and feeding practices in aquaculture (2nd ed., pp. 309–335). Woodhead Publishing.

[vms31461-bib-0096] Vijayakumar, S. , Vaseeharan, B. , Sudhakaran, R. , Jeyakandan, J. , Ramasamy, P. , Sonawane, A. , Padhi, A. , Velusamy, P. , Anbu, P. , & Faggio, C. (2019). Bioinspired zinc oxide nanoparticles using *Lycopersicon esculentum* for antimicrobial and anticancer applications. Journal of Cluster Science, 30(6), 1465–1479. 10.1007/s10876-019-01590-z

[vms31461-bib-0097] Villaró, S. , Ciardi, M. , Morillas‐España, A. , Sánchez‐Zurano, A. , Acién‐Fernández, G. , & Lafarga, T. (2021). Microalgae derived astaxanthin: Research and consumer trends and industrial use as food. Foods, 10(10), 2303. 10.3390/foods10102303 34681351 PMC8534595

[vms31461-bib-0098] Wang, Z. , Cai, C.‐F. , Cao, X.‐M. , Zhu, J.‐M. , He, J. , Wu, P. , & Ye, Y.‐T. (2018). Supplementation of dietary astaxanthin alleviated oxidative damage induced by chronic high pH stress, and enhanced carapace astaxanthin concentration of Chinese mitten crab *Eriocheir sinensis* . Aquaculture, 483, 230–237. 10.1016/j.aquaculture.2017.10.006

[vms31461-bib-0099] Wu, X. , Zhao, L. , Long, X. , Liu, J. , Su, F. , & Cheng, Y. (2017). Effects of dietary supplementation of *Haematococcus pluvialis* powder on gonadal development, coloration and antioxidant capacity of adult male Chinese mitten crab (*Eriocheir sinensis*). Aquaculture Research, 48(10), 5214–5223. 10.1111/are.13333

[vms31461-bib-0100] Xue, X.‐L. , Han, X.‐D. , Li, Y. , Chu, X.‐F. , Miao, W.‐M. , Zhang, J.‐L. , & Fan, S.‐J. (2017). Astaxanthin attenuates total body irradiation‐induced hematopoietic system injury in mice via inhibition of oxidative stress and apoptosis. Stem Cell Research & Therapy, 8(1), 7. 10.1186/s13287-016-0464-3 28115023 PMC5260077

[vms31461-bib-0101] Yano, T. (1992). Assays of hemolytic complement activity. In J. S. Stolen , T. C. Fletcher , D. P. Anderson , S. C. Hattari , & A. F. Rowley (Eds.), Techniques in fish immunology (pp. 131–141.). SOS Publications.

[vms31461-bib-0102] Yin, Y. , Xu, N. , Qin, T. , Zhou, B. , Shi, Y. , Zhao, X. , Ma, B. , Xu, Z. , & Li, C. (2021). Astaxanthin provides antioxidant protection in LPS‐induced dendritic cells for inflammatory control. Marine Drugs, 19(10), 534. 10.3390/md19100534 34677433 PMC8540215

[vms31461-bib-0103] Yu, Y. , Wang, Q. , Huang, Z. , Ding, L. , & Xu, Z. (2020). Immunoglobulins, mucosal immunity and vaccination in teleost fish. Frontiers in Immunology, 11, 567941. 10.3389/fimmu.2020.567941 33123139 PMC7566178

[vms31461-bib-0104] Zhao, X. , Wang, K. , Zhao, J. , Sun, R. , Shang, H. , Sun, C. , Liu, L. , Hou, J. , & Jiang, Z. (2022). Physical and oxidative stability of astaxanthin microcapsules prepared with liposomes. Journal of the Science of Food and Agriculture, 102(11), 4909–4917. 10.1002/jsfa.11854 35246844

[vms31461-bib-0105] Zhu, X.‐M. , Li, M.‐Y. , Liu, X.‐Y. , Xia, C.‐G. , Niu, X.‐T. , Wang, G.‐Q. , & Zhang, D.‐M. (2020). Effects of dietary astaxanthin on growth, blood biochemistry, antioxidant, immune and inflammatory response in lipopolysaccharide‐challenged *Channa argus* . Aquaculture Research, 51(5), 1980–1991. 10.1111/are.14550

[vms31461-bib-0106] Zhuang, Y. , & Zhu, M.‐J. (2021). Recent developments in astaxanthin production from *Phaffia rhodozyma* and its applications. In G. A. Ravishankar , & A. R Rao (Eds.), Global perspectives on astaxanthin (pp. 225–251). Academic Press.

